# Gender Differences in Risk Factor Profile and Clinical Characteristics in 89 Consecutive Cases of Cerebral Venous Thrombosis

**DOI:** 10.3390/jcm10071382

**Published:** 2021-03-30

**Authors:** Zoltan Bajko, Anca Motataianu, Adina Stoian, Laura Barcutean, Sebastian Andone, Smaranda Maier, Iulia-Adela Drăghici, Andrada Cioban, Rodica Balasa

**Affiliations:** 1 Department of Neurology, University of Medicine, Pharmacy, Sciences and Technology Targu Mures, 540136 Targu Mures, Romania; zoltan.bajko@umfst.ro (Z.B.); anca.motataianu@umfst.ro (A.M.); laura.barcutean@umfst.ro (L.B.); smaranda.maier@umfst.ro (S.M.); rodica.balasa@umfst.ro (R.B.); 2 Ist Neurology Clinic, Mures County Clinical Emergency Hospital, Targu Mures, 540136 Targu Mures, Romania; adn.sebastian.007@gmail.com (S.A.); iuliab93@yahoo.com (I.-A.D.); andrada_farcas@yahoo.com (A.C.); 3 Department of Pathophysiology, University of Medicine, Pharmacy, Sciences and Technology Targu Mures, 540136 Targu Mures, Romania

**Keywords:** risk factor profile, cerebral venous thrombosis, gender

## Abstract

Gender has been shown to be an important variable in cerebral venous thrombosis (CVT) risk and significantly influences its clinical manifestations and outcome. The aim of our study was to investigate the gender-specific risk factor profile and clinical picture of this rare cerebrovascular disorder. Materials and methods: We retrospectively reviewed the medical records of 89 consecutive cases of CVT at a tertiary neurology clinic in Târgu Mures, Romania, between June 2009 and January 2021 to analyze the gender-related differences in etiology, clinical presentation, and outcome. Results: Women comprised 62.5% of the cohort. Females were significantly younger than males (37.3 years versus 48.8 years, respectively, *p* = 0.001), and the main risk factors were hormone related in 37.9% of the cases, followed by primary thrombophilia (34.4%), smoking (25.8%), obesity (17.2%), infections (17.2%), mechanical factors (17.2%), cancer (8.6%), systemic autoimmune disorders (8.6%), and hematological disorders (8.6%). In male patients, the main risk factors were smoking (41.9%), primary thrombophilia (29%), infections (22.6%), heavy alcohol consumption (16.1%), and venous thromboembolism in the medical history (12.9%). Frequency of headache was higher in females than in males (75.9% versus 67.7%), whereas frequency of coma (6.5% in males versus 1.7% in females) and dizziness (19.4% in males versus 10.3% in females) was higher in males. CVT onset was acute in 41.4% of females and 38.7% of males. The Rankin score at discharge was significantly lower in females compared with males (0.6 versus 1.6), reflecting a more favorable short-term outcome. Mortality was 6.4% in males and 1.7% in females. Conclusions: CVT is a multifactorial disorder that has a broad spectrum of risk factors with important gender-related differences in clinical manifestation and prognosis. Female patients, especially those with hormone-related risk factors, have a more favorable outcome than male patients.

## 1. Introduction

Gender is an important variable in cerebrovascular and cardiovascular diseases, which is reflected in major differences in the rate and evolution of stroke, myocardial infarction, and venous thromboembolism [1]. The mechanism by which gender functions as an important disease modifier in vascular pathology is not fully elucidated, but there is evidence that sex influences even platelet function and coagulation factor activity [2,3,4,5,6].

Cerebral venous thrombosis (CVT) is a rare but important subtype of stroke comprising 0.5–1% of all stroke cases and, because of protean and non-specific clinical presentation, represents a diagnostic challenge, requiring a high level of clinical awareness for early diagnosis [7]. CVT predominantly affects young adults, with an important female predominance. This uneven gender distribution is mainly attributable to hormone-related risk factors such as oral contraceptive medication, pregnancy, and puerperium and hormone replacement therapy [7,8,9]. The percentage of affected female patients prior to 1981 was 54.8%. This rate significantly increased over time to 69.8% after 2001. This shift is probably attributable to the increase in the use of oral contraceptive medication. The percentage of pregnancy-related cases remained stable [10]. However, newer studies from developed countries reported important changes in the CVT epidemiology in the last decade as significantly higher incidence and less conspicuous gender differences than previously thought. This trend can be explained by the increased availability of high-quality imaging, increased awareness among physicians, and common use of low-estrogen contraceptive medication [11,12,13].

There are limited data in the literature from the Eastern European region regarding gender differences in risk factor profile and clinical characteristics in CVT. The aim of our study was to disclose these aspects by analyzing a cohort of consecutive CVT cases from a tertiary neurology clinic.

## 2. Materials and Methods

We reviewed the medical records of 89 consecutive cases of CVT at a tertiary neurology clinic in Targu Mures, Romania, between June 2009 and January 2021. These cases represented 0.68% of all stroke cases hospitalized in our department. CVT cases were selected from our stroke database based on the discharge diagnosis.

CVT was diagnosed on the basis of either computed tomography venous angiography or magnetic resonance venography, in addition to native and contrast-enhanced computed tomography (CT) or magnetic resonance imaging (MRI).

We documented the detailed risk factor profile, clinical characteristics, and prognosis of all the cases. Severity of CVT was measured using the National Institute of Health Stroke Scale (NIHSS) at admission and modified Rankin scale (mRS) at discharge.

Ethical approval was obtained from the institutional review board of the hospital (31781/07.01.2021).

For statistical analysis we used descriptive methods, expressed as means and standard deviations (SDs), or frequencies and percentages. Differences between groups were described using standard statistics, evaluated for significance using two-sample *t*-tests or the Mann–Whitney *U* test. The categorical data were analyzed using Fisher’s exact test.

We compared the male and female patient groups, in addition to the female subgroups—with and without hormone-related risk factors.

## 3. Results

Of the 89 included patients, 58 were female (65.2%). Female patients were significantly younger than male patients (37.3 ± 14.5 years versus 48.8 ± 15.6 years, *p* = 0.001).

### 3.1. Risk Factor Profile

Table 1 presents the main risk factor profile in the male and female groups.

The main primary prothrombotic conditions in the whole cohort in order of frequency were Factor V Leiden mutation, hyperhomocysteinemia, protein C or S deficiency, antithrombin III deficiency, and prothrombin gene mutation, without gender differences.

### 3.2. Characteristics of Thrombosis

There was no significant difference between males and females regarding the distribution of the occluded veins and sinuses. Multiple veins were affected in more than 50% of both groups. The treatment regimens were similar; all the patients received low-molecular-weight heparin (LMWH) followed by oral anticoagulation. The majority of patients received vitamin K antagonists (VKA) (92.1%). Only a minority of the cases received non-anti vitamin K anticoagulants (NOAC) for chronic oral anticoagulation (5.2% of females, 12.9% of males). None of the patients underwent endovascular treatment or decompressive surgery.

The frequency of headache was higher in females than in males (75.9% versus 67.7%, respectively), whereas the frequency of coma (6.5% in males versus 1.7% in females) and dizziness (19.4% in males versus 10.3% in females) was higher in males than in females. Male patients with coma were older (76 and 53 years) and presented multiple venous affection and extensive hemorrhagic lesions. The female patient with coma was younger (28 years) and presented bilateral deep cerebral vein thrombosis, and the main risk factors were the primary hypercoagulability (Factor V Leiden mutation) and contraceptive medication (Table 2).

There were no significant differences regarding the laboratory analysis, with the exception of the hemoglobin and hematocrit values, which were significantly lower in females (*p* = 0.0001). This is attributable to the blood loss during labor and cesarean delivery in the postpartum patient group (Table 2).

### 3.3. Outcomes

The National Institute of Health Stroke Scale (NIHSS) value was similar in the two groups, but the Rankin score at discharge was significantly lower in females than in males (0.6 ± 1.1 versus 1.6 ± 1.7, respectively), reflecting a more favorable short-term outcome. Mortality was 6.4% in males and 1.7% in females (Table 2).

### 3.4. Characteristics of Female Subgroups

We analyzed the two female subgroups separately: females without hormone-related risk factors (*n* = 36) and females with hormone-related risk factors (*n* = 22).

The mean age of the female group without hormone-related risk factors did not differ significantly from that of the male group (42.4 years versus 48.8 years, respectively), but the patients with hormone-related risk factors were significantly younger (28.8 years, *p* < 0.0001).

The frequency of primary thrombophilia was the highest in the female group with hormone-related risk factors. Cancer, systemic autoimmune disorders, hematological disorders, and infection were more frequent in the female group without hormone-related risk factors. The frequency of lumbar puncture as a mechanical factor was the highest in the female group with hormone-related risk factors.

Obesity was more frequent in both female groups compared with in males. The frequency of smoking and heavy alcohol consumption was the highest in the male group (41.9% and 16.1%, respectively); however, the frequency of smoking was also high in both female groups (≥25%). The frequency of venous thromboembolism in the medical history was the highest in the male group (12.9%). NIHSS did not differed significantly between the three groups, but the mRS at discharge was lower in both female groups compared with males, reflecting a more favorable outcome in females.

The mortality was zero in the patient group with hormone-related risk factors (Table 3).

## 4. Discussion

Our single center study including a more then 10 year-cohort of consecutive patients from a tertiary hospital demonstrated several gender differences in the CVT risk factor profile, clinical characteristics, and short-term outcome. The main differences in the risk factor profile are attributable to the gender-related risk factors such as oral contraceptive medication use and pregnancy/puerperium. However, there are differences regarding lifestyle-related risk factors, such as smoking and heavy alcohol consumption being more frequent in males, and obesity in females. Analyzing separately the two female subgroups (with and without hormone related risk factors), we observed that between the female group without hormone-related risk factors and male group the age difference disappeared. In contrast, important differences remained regarding the incidence of cancer, autoimmune disorders, hematological disorders, and obesity, which were more frequent in females; and smoking, heavy alcohol consumption, and venous thromboembolism, which were more frequent in the medical history in males. The most striking difference between groups was the significantly more favorable outcome in both female groups compared with males.

In our cohort there were no severe cases requiring invasive or surgical management for elevated intracranial pressure. In larger cohorts, even female patients with combined hormonal contraceptive medication developed severe intracranial hypertension requiring invasive interventions that involve a lower complication rate and better outcome than conservative treatment such as barbiturate coma or induced hypothermia [14].

Few studies in the literature have purposefully investigated gender differences in CVT. Coutinho et al. [8] published a study in 2009 on the characteristics of CVT in women using a large prospective multicenter international database. The study included data from 624 patients, 75% of whom were female. The gender-specific risk factors were present in a very high percentage in women compared with our cohort (65% versus 37.9%). This difference is attributable mainly to the lower rate of oral contraceptive usage in our patients (only 20.7% versus 46%); this data reflects the lower rate of oral contraception in our geographical region. Regarding the clinical manifestations, in their study only headache was significantly more frequent in female patients; this was observed also in our cohort, but without statistical significance. The mean age of the females with hormone-related risk factors was very similar to that in our cohort (29 years), but our male patients were older (48.8 versus 42). In line with our results, they also found significantly better outcome in females with hormone-related risk factors, irrespective of other risk factors. The main explanations for better outcome in females with hormone-related risk factors may be, in addition to the younger age, their better general medical condition and more frequent medical visits due to the oral contraceptive medication prescriptions and pregnancy follow-up [8,15,16].

Hinnell et al. published a single-center retrospective study in 2012 with very similar patient number and time range as in our study. Of the 108 consecutive patients, 62% were female. The rate of oral contraceptive users was more than double (45%) that of our data (20.7%). The frequency of obesity was higher in their cohort in all subgroups, and the frequency of smoking and primary thrombophilia was lower. These results probably reflect the epidemiological differences regarding obesity and smoking in the general population between different geographical regions. In line with our results, the female patients with hormone-related risk factors presented less residual focal neurological deficit compared with the other groups [9].

Hormonal contraception was also an important risk factor in our cohort. In a meta-analysis published by Amoozegar et al. there was a sevenfold increase in risk of CVT in patients using oral contraceptive medication [17]. The combination of oral contraceptive medication and genetic thrombophilia significantly increases the risk of CVT, especially in women with factor V Leiden mutation, prothrombin gene mutation, and hyperhomocysteinemia [17,18,19,20]. Pomp et al. demonstrated that women who were currently smokers and on oral contraceptive medication had an 8.8-fold higher risk of venous thrombosis than nonsmoking women without oral contraceptives [21].

In our cohort the obesity was most frequent in the female group without hormone-related risk factors (19.4% vs. 9.7% in males). In a case-control study published by Zuurbier et al., obesity was associated with increased risk of CVT in women, but not in men. The association was stronger in women on oral anticontraceptive medication [22].

There are limited data in the literature regarding alcohol consumption and CVT risk. In our cohort the rate of heavy alcohol consmption was 16.1% in the male group. According to the results of a meta-analysis published by Green et al., alcohol consumption increases the risk of CVT by 2.67-fold. Interestingly, hypertension, diabetes, and especially smoking were not associated with CVT risk [23]. However, another systematic review investigating the risk of venous thromboembolism in smokers revealed that current smoking and former smoking status are associated with increased venous thromboembolism risk with a dose–response relationship [24].

An important part of our female patient group was in the puerperium (17.2%).The postpartum period represents the highest risk of CVT in pregnancy-related cases. The main reason for this association is the disequilibrium of the coagulation system that during the pregnancy presents a shift from fibrinolysis to enhanced hemostasis to control hemorrhage during labor. These changes return to baseline only 6–8 weeks after delivery. Other factors that enhance thrombotic risk are added to the above modifications, such as cesarean delivery, anemia secondary to blood loss, dehydration, intracranial pressure fluctuations during labor, pre-eclampsia and eclampsia, and the dural puncture during spinal analgesia [25,26,27,28,29,30,31]. The loss of cerebrospinal fluid after dural puncture may lead to decreased intracranial pressure, compensatory venous and arterial dilation, decreased venous flow, endothelial damage, and activation of the coagulation system [26]. Silvis et al. demonstrated that women after delivery present an increased risk of CVT, but not during pregnancy [32].

In line with the literature data, our female patient group without hormone-related risk factors presented the highest rate of cancer. Neoplastic disorders can increase the CVT risk in different ways. Systemic thrombosis is well known in this setting, secondary to cancer-related hypercoagulability, especially in different adenocarcinomas. The direct effect of tumors via compressive effect or carcinomatous infiltration, infections secondary to immunosuppression, and the side effects of therapeutical measures, especially systemic chemotherapy, can also lead to increased thrombotic risk [33].

The incidence of systemic inflammatory disorders was also highest in females without hormone-related risk factors. The most frequent in our cohort was systemic lupus erythematosus. According to some case reports, CVT can even be the first manifestation of lupus [34].

The frequency of local infections as a risk factor was higher in our male group, consistent with the literature data [8]. Otitis and mastoiditis may lead to thrombosis of the adjacent lateral sinuses. Cavernous sinus thrombosis can be a complication of an infection of the middle third of the face, paranasal sinuses, or dental infections. SSS thrombosis can be a result of sinusitis or meningeal infection [35].

The frequency of venous thromboembolism in the medical history of our cohort was higher in our male patients than in our female patients. According to literature data, the recurrence of thrombosis after the first CVT event is also higher in males. Lym et al. investigated the risk of thrombotic recurrence in 52 CVT cases over a 10.5-year period in Northeast Melbourne. During the follow-up period they found three symptomatic thrombotic recurrences (one CVT, one portal vein thrombosis, one inferior vena cava thrombosis) [36].

The main limitation of our study is its retrospective nature and relatively small number of patients that arises from the single-center design.

In conclusion, our results indicate that CVT is a multifactorial disorder with a broad spectrum of risk factors. There are important gender-related differences in the risk factor profile, clinical presentation, and outcome of CVT. Female patients, especially those with hormone-related risk factors, have a more favorable outcome compared with males. Further studies are required to elucidate the background of this association in order to implement gender-specific efficient preventive measures.

## Figures and Tables

**Table 1:** Risk factor profile in male and female groups.

Risk Factors		Male Group (Nr. of Cases 31)	Female Group (Nr. of Cases 58)
Primary thrombophilia		9 (29.0%)	20 (34.4%)
Women’s health conditions	All	0	22 (37.9%)
	Hormonal contraception	0	12 (20.7%)
	Hormone replacement therapy	0	0
	Pregnancy	0	0
	Puerperium	0	10 (17.2%)
Cancer		1 (3.2%) (pulmonary)	5 (8.6%) (1 ENT, 1 gynecological, 1 breast)
Systemic autoimmune disorders		1 (3.2%) lupus	5 (8.6%) (3 lupus 2 autoimmune thyroid disease)
Hematological disorders		1 (3.2%) thrombocytosis	5 (8.6%) 1 thrombocytosis 1 pancytopenia 1 paroxysmal nocturnal hemoglobinuria 1 Hodgkin, 1 Non-Hodgkin lymphoma
Infection	All infections	7 (22.6%)	10 (17.2%)
	Local infections (ear, sinus, face, neck)	5 (16.1%)	7 (12.1%)
	Systemic infection-sepsis	1 (3.2%)	3 (5.2%)
	Meningeal infection	1 (3.2%) (meningitis)	0
Mechanical factors	All	1 (3.2%)	10 (17.2%)
	Neurosurgical intervention	0	3 (5.2%)
	Lumbar puncture	0	6 (10.3%)
	Head trauma	1 (3.2%)	1 (1.72%)
Obesity		3 (9.7%)	10 (17.2%)
Smoking		13 (41.9%)	15 (25.8%)
Heavy alcohol consumption		5 (16.1%)	2 (3.4%)
Venous thromboembolism in the medical history		4 (12.9%)	2 (3.4%)
No risk factor identified		9 (29.0%)	7 (12.1%)

**Table 2:** The main clinical and paraclinical characteristics in male and female groups.

		Male (Nr. of Cases 31)	Female (Nr. of Cases 58)	
Affected sinuses	Multiple veins affected	19 (61.3%)	31 (53.4%)	
	Single vein affected	12 (38.7%)	27 (46.5%)	
	Superior sagittal sinus	19 (61.3%)	25 (43.1%)	
	Lateral sinuses (transverse and/or sigmoid)	22 (70.9%)	40 (68.9%)	
	Inferior sagittal, Straight sinus	1 (3.2%)	7 (12.1%)	
	Deep veins	1 (3.2%)	3 (5.2%)	
	Cortical veins	2 (6.5%)	5 (8.6%)	
	Cerebellar veins	1 (3.2%)	0 (0%)	
	Jugular veins	1 (3.2%)	2 (3.4%)	
	Cavernous sinus	2 (6.5%)	1 (1.7%)	
Clinical manifestations	Headache	21 (67.7%)	44 (75.9%)	
	Seizures	14 (45.2%)	25 (43.1%)	
	Hemiparesis	7 (22.6%)	12 (20.7%)	
	Aphasia	3 (9.7%)	5 (8.6%)	
	Coma	2 (6.5%)	1 (1.7%)	
	Visual disturbances	1 (3.2%)	3 (5.2%)	
	Encephalopathy/mental status disorder	3 (9.7%)	5 (8.6%)	
	Oculomotor palsies	1 (3.2%)	4 (6.9%)	
	Dizziness	6 (19.4%)	6 (10.3%)	
	Nausea, vomiting	10 (32.3%)	18 (31.0%)	
The mode of onset	Acute (<48 h)	12 (38.7%)	24 (41.4%)	
	Subacute (>48 h to 30 days)	18 (58.1%)	31 (53.4%)	
	Chronic (>30 days)	1 (3.2%)	3 (5.2%)	
Parenchymal lesion on imaging	Hemorrhagic lesion	9 (29.0%)	12 (20.7%)	
	Nonhemorrhagic lesion (edema, infarction)	17 (54.8%)	30 (51.7%)	
				*p* value
Age		48.8 ± 15.6	37.3 ± 14.5	<0.001
NIHSS at admission		3.4 ± 5.6	3.1 ± 5.4	0.76
Rankin Scale at discharge		1.6 ± 1.7	0.6 ± 1.1	0.009
D-dimer (μg/mL)		2.3 ± 2.1	3.1 ± 1.9	0.41
Cholesterol (mg/dL)		191.8 ± 49.8	197 ± 46.2	0.6
Triglyceride (mg/dL)		167.4 ± 87.9	169.3 ± 138.4	0.9
Hemoglobin (g/dL)		14.6 ± 1.8	13.2 ± 1.6	0.0001
Hematocrit (%)		44.6 ± 5.1	38.4 ± 6.9	<0.0001
Thrombocyte (/mm^3^)		234,485.7 ± 105,192	259,234.9 ± 86,051	0.07
Leukocyte (/mm^3^)		9706.7 ± 3486.2	8378.8 ± 2750.6	0.28
ESR (mm/h)		20.3 ± 20.0	22.5 ± 16.9	0.19
Mortality		2 (6.4%)	1(1.7%)	NS

NS: not significant; NIHSS: National Institute of Health Stroke Scale; ESR: erythrocyte sedimentation rate.

**Table 3:** Risk factor profile and clinical characteristics in male group and female subgroups: females without hormone-related risk factors and females with hormone-related risk factors.

Risk Factors		A. Male Group (Nr. of Cases 31)	B. Female Group without Hormone-Related Risk Factors (Nr. of Cases 36)	C. Female Group with Hormone-Related Risk Factors (Nr. of Cases 22)	*p*-Value (A–B)	*p*-Value (A–C)	*p*-Value (B–C)
Age		48.8 ± 15.6	42.4 ± 16.1	28.8 ± 6.1	NS	<0.0001	<0.0001
Primary thrombophilia		9 (29.0%)	11 (30.5%)	9 (40.1%)	NS	NS	NS
Cancer		1 (3.2%) (pulmonary)	3 (8.3%) (1 ENT, 1 gynecological, 1 breast)	0	NS	NS	NS
Systemic autoimmune disorders		1 (3.2%) lupus	3 (8.3%) (1 lupus 2 autoimmune thyroid disease)	2 (9.1%) (2 lupus)	NS	NS	NS
Hematological disorders		1 (3.2%) thrombocytosis	5 (13.8%) 1 thrombocytosis 1 pancytopenia 1 paroxysmal nocturnal hemoglobinuria 1 Hodgkin, 1 Non-Hodgkin lymphoma	0	NS	NS	NS
Infection	All infections	7 (22.6%)	8 (25%)	2 (9.1%)	NS	NS	NS
	Local infections (ear, sinus, face, neck)	5 (16.1%)	6 (16.6%)	1 (4.5%)	NS	NS	NS
	Systemic infection-sepsis	1 (3.2%)	2 (5.5%)	1 (4.5%)	NS	NS	NS
	Meningeal infection	1 (3.2%) (meningitis)	0	0	NS	NS	NS
Mechanical factors	All	1 (3.2%)	5 (13.8%)	5 (22.7%)	NS	NS	NS
	Neurosurgical intervention	0	3 (8.3%)	0	NS	NS	NS
	Lumbar puncture	0	1 (2.7%)	5 (22.7%)	NS	0.01	NS
	Head trauma	1 (3.2%)	1 (2.7%)	0	NS	NS	NS
Obesity		3 (9.7%)	7 (19.4%)	3 (13.6%)	NS	NS	NS
Smoking		13 (41.9%)	9 (25%)	6 (27.2%)	NS	NS	NS
Heavy alcohol consumption		5 (16.1%)	2 (5.5%)	0	NS	NS	NS
Venous thromboembolism in the medical history		4 (12.9%)	1 (2.7%)	1 (4.5%)	NS	NS	NS
No risk factor identified		9 (29.0%)	7 (19.4%)	0	NS	0.02	NS
NIHSS at admission		3.4±5.6	3.7±5.9	2±4.1	NS	NS	NS
Rankin Scale at discharge		1.6±1.7	0.8±1.3	0.4±0.8	0.04	0.001	NS
Mortality		2 (6.4%)	1 (2.7%)	0%	NS	NS	NS

NS: not significant; ENT: ear nose throat; NIHSS: National Institute of Health Stroke Scale.

## Data Availability

Data isavailable from the corresponding author on reasonable request.
